# The Veterinary Service of the United States Army

**Published:** 1887-04

**Authors:** R. W. Shufeldt

**Affiliations:** Captain, Medical Dept. U. S. Army


### Art. XIII.—THE VETERINARY SERVICE OF THE UNITED STATES ARMY.

BY R. W. SHUFELDT, M. D., C. M. Z. S., ETC.
*Captain, Medical Dept. U. S. Army.*

Some fourteen or fifteen years ago I attended the admirable course of lectures upon veterinary medicine and surgery, including the anatomy of the domesticated animals, delivered at Cornell University, by Professor James Law. Since that time I have seen more or less of the methods adopted to care for the sick and disabled horses of the Army. My interest in this matter has also been aroused by articles that have appeared from time to time in THE JOURNAL dealing with the various phases of this subject.

A letter which I read in a foreign periodical a week or ten days ago, has induced me to ask your indulgence in the matter of a little space, to add a few words of my own to this very important question.

It will not be my object in this paper, however, to offer any detailed account of the present organization of the veterinary service in our Army, as its status, and its highly unsatisfactory condition, are already too well known to us. As we are aware, our few veterinary surgeons attached to the cavalry regiments are not even commissioned officers; nor is their position as desirable as some of the positions in the non-commissioned staff; nor have they the requisite authority that should pertain to the Army veterinary surgeon; further, their facilities for taking the proper care of sick and disabled government horses are, to say the least of it, simply wretched. In short, the vast majority of the diseased and disabled animals fall into the hands of troop farriers and blacksmiths—men of little or no education, and of no special training in veterinary science whatever.

The former class of men are far too indifferently paid to insure the securing of persons of recognized ability, while the latter should simply constitute the attendants of the department.

It is a rare thing to find a cavalry officer, or an officer of a light battery, who has any *real* knowledge of such subjects, and somehow they seem to fail to appreciate the importance of improving the present state of affairs. Occasionally we will find a medical officer of the army who will *condescend* to go and see an injured government animal, but there are even exceptions to this rule; and one will sometimes say, "No, sir, I consider it far beneath my dignity to know anything of any such subject, I am commissioned for a far higher office." Ah, can it be beneath anyone's "dignity" to be found familiar with any *fact* so long as it represents true knowledge?

I have accepted invitations a number of times during the past ten years from veterinary surgeons of the army to witness operations upon horses, and I must confess that for the most part, the entire scene has usually had the effect of profoundly disturbing my rest for the succeeding night by a hideous night *mare.*

At one of the frontier military posts in Wyoming there was convened, during the summer of 1879, a board of officers to decide what disposition they would recommend to be made of a valuable government horse which had been stricken down in one of the post cavalry stables with idiopathic tetanus. I was present by invitation at the time, and was incidently asked if any thing could be done for the animal, and I recollect giving it as my opinion, that the disease usually proved fatal, but would suggest the trial of removing the patient to a quiet, roomy building, where it could be supplied and protected by a thick bed of straw, and to administer a full dose of aloes, to be followed by belladonna, and then suggested briefly what the subsequent treatment should be. The dose of aloes was administered in my presence, but in the mean time the board had further consulted together, and I saw in a few moments coming in another direction the "subsequent treatment"—a loaded government carbine, calibre 45. This latter instrument is the one most frequently called into use in nearly all severe cases among the horses and mules of our army, and it has one especial advantage—that of covering up a deal of ignorance, to say nothing else.

So far as my experience goes I have never seen a fracture in a government horse that was treated in any other way than by further complicating the case by a fatal gun-shot wound of the head, administered at the recommendation of a "board of survey." The *carbine* is also frequently the sole treatment in many of the other surgical injuries, and in the more obscure diseases.

It will not require figures and facts here, to show that a properly organized veterinary department for the army would be a far wiser, a better and a more economical arrrangement than the present management. Lack of space alone, however, will prevent me from making the necessary comparisons here to demonstrate it.

Were I to draw up a plan for improvement in that direction, it would be something of the following nature:

In the first place it should be organized strictly upon the basis of a staff department, and for its organization it could borrow a great many valuable suggestions from the Medical Department of the Army, and more especially those regulations which have been found to operate to the best ends in actual practice. Right here, however, let me call attention to one fatal error, now in force in the Medical Department, which a veterinary corps should most strenuously avoid, and that is—down through the very lowest subordinate of the department they should, one and all, be actually members of it, and not be obliged thoroughout the service to have men detailed from troops, companies or batteries, to do the duties of the department, duties of which they, in the vast majority of cases, are grossly ignorant.

The veterinary department should consist of a limited number of commissioned officers, a non-commissioned staff, sufficiently large to properly assist in performing the duties pertaining to it, and, finally, a corp of attendants.

Fully as much care should be exercised in the selection of the officers as if they were being chosen for one of the best corps connected with the service. They should be cultured and refined gentlemen in every sense of the word. Further, they should be picked graduates of a limited number of the best recognized veterinary colleges, either of this country, Canada, or Europe. They should have the rank, pay and emoluments of officers of cavalry; they should be subject to promotion within their own department; and they should be allowed the usual increase of pay for length of service. The chief of the department should be a major; there should be three captains, and a requisite number of first and second lieutenants.

Non-commissioned officers, with duties comparable with those of the present class of hospital stewards in the army, could be chosen by a board of veterinary officers, and, if necessary, from the army, provided they could meet the requirements of the board; veterinary attendants could be secured in the same way.

There are a great many valuable men in the army to-day who would make excellent attendants, were they permanently attached to a veterinary department and fell under the jurisdiction of veterinary officers, capable of disciplining and instructing them.

The day is rapidly approaching when an entire regiment of cavalry will be stationed at one point, and these points will naturally be selected for cavalry service, and here will be the localities to establish regimental veterinary hospitals. Troops detached could have a veterinary non-commissioned officer detailed with them, and be subject to periodical visits from a commissioned officer of the department at stated times.

The department should be supplied in much the same way as the medical department is at present, and should, moreover, have the same authority over sick and disabled government animals, as the medical department has over the sick and disabled of the army. In short, after such a veterinary department was once established, it could be conducted pretty much on the same plan as the medical department is at present, and subjected to very much the same kind of regulations.

A small department like this, properly organized and equipped, and in successful operation in peace times, would form an invaluable nucleus upon which to build in times of war. There cannot be too much said in favor of our establishing such nuclei in our military services during these days. But after all, it would be during the long days of peace in the country that the greatest amount of good would accrue from such an establishment.

The class of officers chosen for it that I have suggested would immediately raise the department to a standard that it should occupy, and the mounted arms of the service would at once commence to realize the benefits. Not only would government animals be properly cared for in every particular, but the recruiting of horses and mules could be scientifically attended to by the veterinary department, and thus a far better class of material with greater certainty be secured. Medical and surgical cases, and epidemics could be carefully studied and exhaustively reported upon by the head of the department, incorporating the reports of special cases of individual officers. And these latter coming from the best class of veterinary colleges, would be fully imbued with the great importance of making *comparative* studies in the diseases and injuries among *all* the domesticated animals; they would, too, be inclined to point out the relationship or differences of these diseases, with such diseases as are known to afflict mankind. In fact they would probably be a class of men who would fully recognize what we are just beginning to recognize in these days—and that is to really know what pathology is, and its great scope; we *must* study it in every organized structure that it is known to invade, and then make exhaustive comparative studies of the whole. These officers, too, would probably have a larger share of leisure time upon their hands than their professional brethren in active veterinary practice in the large cities. They could thus inaugurate, when opportunity offered, careful investigations into fatal epidemics among cattle, and sheep, and swine; they would be enabled to make long and careful studies of normal structure, and when serving in certain districts in the west, could more fully develop a *now sadly neglected field*, the morphology and physiology of our United States mammalian fauna. Many of our wild animals in the territories are rapidly being exterminated under our very eyes, and in a few years not only will these important types be forever gone, but we will have the deplorable fact forced upon us, that we have allowed them to disappear and without, in many instances, leaving us, as a legacy, a single line, or a single drawing illustrating their structure.

Following the organization of such a corps as I here propose, not only would the government and the army be well served, but science would also claim her share, and thus humanity at large be well served, and veterinary science in particular would be the gainer; indeed, space will not allow me to fully enumerate the immense advantages that would follow in the wake of such an establishment; and I trust, the day ever arrives when man himself does not constitute the greatest obstacle to his own progress, such a government veterinary corps will duly become a living reality.

